# Numerical modeling of radionuclide migration through a borehole disposal site

**DOI:** 10.1186/2193-1801-3-155

**Published:** 2014-03-21

**Authors:** Serwaa Yeboah, Thomas T Akiti, John J Fletcher

**Affiliations:** Graduate School of Nuclear and Allied Sciences, Atomic Energy, University of Ghana, P.O. Box AE 1, Legon, Ghana; National Radioactive Waste Management Centre, National Nuclear Research Institute, Ghana Atomic Energy Commission, Box LG 80, Legon, Ghana

**Keywords:** Migration, Borehole repository, Heterogeneity, Simulation, Numerical model, Comsol Multiphysics

## Abstract

The migration of radionuclides from a borehole repository located about 20 km from the Akwapim fault line which lies in an area of high seismicity was analyzed for some selected radionuclides. In the event of a seismic activity, fractures and faults could be rejuvenated or initiated resulting in container failure leading to the release of radionuclides. A numerical model was solved using a two-dimensional finite element code (Comsol Multiphysics) by taking into account the effect of heterogeneities. Results showed that, the fractured medium created preferential pathways indicating that, fault zones generated potential paths for released radionuclides from a radioactive waste repository. The results obtained showed that variations in hydraulic conductivity as a result of the heterogeneity considered within the domain significantly affected the direction of flow.

## Introduction

**Figure 1:**
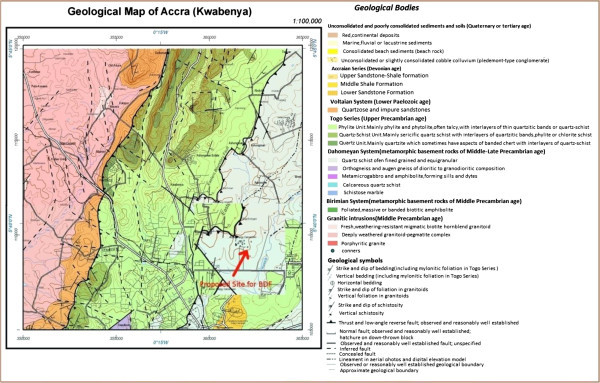
**Geological map of the region immediately around the proposed site.**

The site for the Borehole Disposal Facility is within the Accra Plains and is situated on the boundary between the Dahomeyan System and the Togo Series (Figure 1), both of which are of Precambrian age. The Dahomeyan is the major bedrock formation underlying the site and consists of gneiss and schist while the Togo Series are predominantly composed of quartzite and phyllite. The Akwapim hills and the Togo range mark the line of a major active fault zone that runs North-East and South-West to the coastal boundary fault (Muff & Efa 2006).

The whole of the site is covered by loose unconsolidated and weathered material that may reflect the presence of troughs formed by down faulted blocks which indicates the existence of seismic activity in the geologic past and it probably results from movements along the Akwapim fault line (Junner & Bates 1995).

The only major river near the Borehole Disposal Facility (BDF) site is River Onyasia, located about 1.3 km from the proposed facility and drains southwards through Achimota village to Accra. The Onyasia River has a depth of 0.6 m, a width of 6.8 m and a measured velocity of 0.8 m/s (2.5 × 10^7^ m/y). The broad valley of the Onyasia River flanks the site on its eastern margin with swampy conditions generally found north-east of the site. Surface run-off in this area is very low, however, after heavy storms there is flow of water over the horizon below the top-soil (Darko et al. 1995).

Seismic surveys conducted in the area mapped out two weak lines suspected to have been caused as a result of faults or fractures. However, these have not been used in numerical modelling to follow and predict the migration of radionuclides at the site in case a seismic accident occurs (Essel et al. 2011).

## Methodology

### Model definition

**Figure 2:**
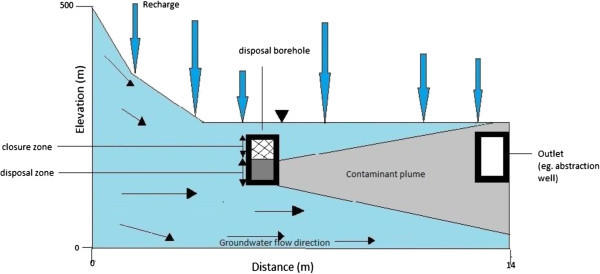
**Conceptual model.**

The hydrogeologic setting for groundwater flow at steady state shall be described by the conceptual model shown in Figure 2. Groundwater moves from a higher topographic surface to a lower topographic surface. The water table is a free surface across which there is vertical recharge, denoted by R. The base of the aquifer is considered impermeable.

### Scenario development

In order to predict radionuclide release, the engineering barriers are assumed to fail in the event of a seismic activity. The system is thus, simplified into a two-dimensional conceptual model as shown in Figure 2.

The radionuclides are initially confined in the canister until a seismic activity occurs, leading to a crack of the barrier system such that the radionuclide inventory is released into the groundwater which is the major transport medium. It is assumed in the calculations performed that radionuclides start being released from the canister 30 years after closure of the repository.

### Numerical illustrations

A two-dimensional numerical model was developed using Comsol Multiphysics (ver.3.4) similar to the proposed model in Figure 2. The lithology of the system was characterized by a porosity of 0.35, a transverse dispersivity of 0.005 m, a longitudinal dispersivity of 0.5 m and a hydraulic conductivity ranging from 10^-15^ to 10^-5^ m/s.

The time-dependent solute transport equation as described by equation (1):1

Here, the dispersion tensor *D*_*L*_ defines solute spreading by mechanical mixing and molecular diffusion. Equations for the tensor entries are:2

Where *D*_*ii*_*, D*_*jj*_ are the principal components of the dispersion tensor based on the Darcy’s velocity, *D*_*ij*_ and *D*_*ji*_ are the cross terms of the dispersion tensor, the subscript “L” denotes longitudinal dispersivity, “T” the transverse dispersivity. *v* is the magnitude of the Darcy’s velocity vector, *D*_*m*_ represents the effective molecular diffusion in a saturated porous media and *i, j* are the spatial coordinates.

## Governing equations

### Fluid flow: assumptions

- ▪ On the scale simulated, the fracture system behaves as an equivalent porous medium
- ▪ The groundwater flow is assumed to be homogenous and subject to recharge
- ▪ Additionally, groundwater flows under steady state conditions. This means that, the velocity of flow is considered not to change with time since groundwater flow is naturally a slow process.

### Fluid flow: domain equations and boundary conditions

Steady groundwater flow is expressed with a conservation equation formulated with Darcy’s law and expressed as:3

Where *K* is hydraulic conductivity (*m/s*); *v*_*i*_ is the Darcy velocity (*m/s*); *x*_*i*_ is the spatial distance (*m*) and *H* is the hydraulic head (*m*); Hydraulic head is a function of pressure and gravitational potential and it is defined:4

Where, *h*_*p*_ is pressure head (*m*); and *y* is elevation (*m*). The equations for groundwater flow and solute transport are linked by:5

Where *R* is the recharge rate (*m*/*s*).

**Figure 3:**
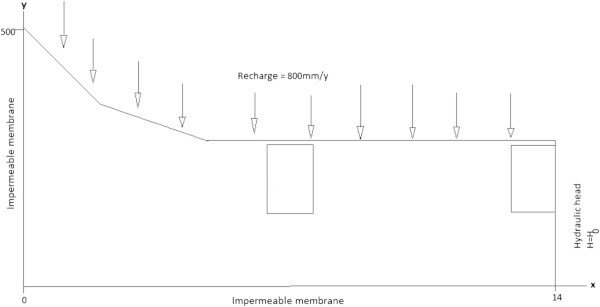
**Boundary conditions for groundwater flow.**

Figure 3 shows the boundary conditions for the groundwater flow problem and stated by equations (6–8).

A zero flux Neumann condition represents the symmetry boundary at *x* = 0 m and the impermeable boundary at *y* = 0 m as follows:6

Hydraulic head is specified at *x* = 14 m with a Dirichlet condition:7

The annual average precipitation rate is recorded as 800 mm/y equivalent to 2.537 × 10^-8^ m/s. The water table receives about 10% of this value. From a topographic height of *y* = 500, an inflow boundary condition that satisfies Neumann’s condition is assumed and is written in the form:8

### Solute transport: assumptions

Transport of radionuclides is based on the following assumptions:

▪ Radioactive decay is the only reaction considered in the model. It is assumed to occur throughout the model in the liquid phase;

▪ For the purposes of this work, no gaseous release is considered;

▪ Transport of radionuclides is assumed to occur in the saturated zone;

### Solute transport: domain equations and boundary conditions

**Figure 4:**
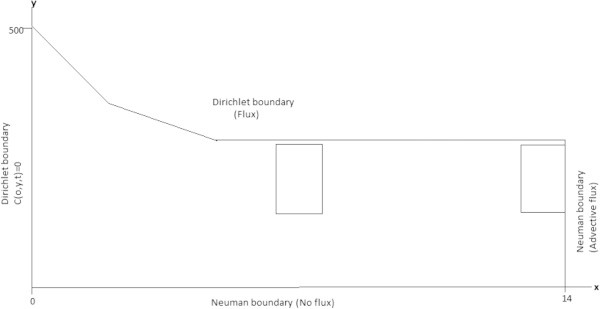
**Solute transport.**

For the transport equation, the initial and boundary conditions for solute transport are shown in Figure 4. In this work, it is also assumed that the contaminant source is continuous over a period of thirty years. Dirichlet conditions are used at the water table, where *C*(*x*, *h*, *t*) = 0, except for the segment 4*m* ≤ *x* ≤ 8*m* in which:9a9b

Where *C*_0_ is the relative concentration through year 30(*t*_0_). The Dirichlet condition at the left boundary is:10

The Neumann condition defining the zero flow boundaries is:11

Finally, the initial condition indicating that the subsurface on the site is free of contaminants at the beginning of the simulation is:12

## Results and discussions

Numerical simulations were considered for Cobalt-60 (short half-life), Cesium-137 (medium half-life) and Americium-241 (long half-life) migrating from the repository through the subsurface environment for a 2-dimensional cross-section along the streamlines of groundwater flow. These considerations were made because of the high amount of activity contained in the waste packages and the variability of their half-life as shown in Table 1.

**Table 1:** **Ghana’s inventory of disused sources** (Essel et al. 2011)

Disused low dose sources				
Radionuclide	Total initial activity (Bq)	Application	Form	Unit pieces
Cs-137	5.66 × 10^12^	Level gauges	Sealed	30
Co-60	1.75 × 10^6^	Non-Destructive Testing (NDT)	Sealed	2
Cs-137/Co-60	4.09 × 10°/4.90 × 10^-1^	-	Sealed	2
Cs-137/Am-241	3.70 × 10^11^/1.85 × 10^12^	-	Sealed	3
Cs-137/Am 241:Be	3.00 × 10^1^/ 1.80 × 10^3^	Nuclear gauges	Sealed	1
Am-241	3.50 × 10^1^	Smoke detectors	Sealed	105
Sr-90	1.25 × 10^4^	Thickness gauges	Sealed	33
Ir-192	2.26 × 10^6^	NDT	Sealed	1
Cd-109	6.66 × 10^2^	Research	Sealed	6
Am-241	1.67 × 10^3^	Nuclear gauge	Sealed	1
I-131	6.21 × 10^9^	-	Unsealed	2
Cf-252	2.22 × 10^10^	-	Sealed	2
Ra-226	7.03 × 10^9^	-	Sealed	19 needles
H-3	3.70 × 10^7^	Nuclear gauge	Unsealed (liquid)	2750 L
C-14	2.60 × 10^7^	-	Unsealed (gas)	7empty cylinders
**Disused high dose sources**				
Co-60	2.78 × 10^8^	Gamma cell-research	Sealed	1
Co-60	1.85 × 10^8^	Teletherapy	Sealed	1
Co-60	2.22 × 10^8^	Food irradiator	Sealed	1
I-129*	4.25 × 10^10^	-	sealed	1
Fe-59	2.22 × 10^4^	-	sealed	2
Co-57	1.11 × 10^2^	-	sealed	3
Zn-65	n3.70 × 10^2^	-	sealed	1
Sr-89	4.77 × 10^3^	-	sealed	1
Tl-204	7.40 × 10^-1^	-	sealed	2
P-32	1.18 × 10^3^	Research	Unsealed	4
S-35	9.25 × 10^-6^/ml	-	Unsealed	5
Ca-45	1.85 × 10^2^	-	Unsealed	3
Na-22	3.7 × 10°	-	Unsealed	4
In-113 m	2.22 × 10^3^	-	Unsealed	12

### Computer simulations

The models describe the steady-state fluid flow and follows up with a transient solute transport simulation. Two partial differential equations (PDE) were solved for and these were assigned in separate mathematics interfaces in Comsol Multiphysics (version 3.4). The first partial differential equation (PDE) is stationary and it finds a solution to the Darcy velocity while the second partial differential equation (PDE) is time-dependent and finds a solution to the solute transport equation.

To test the effect of heterogeneity on solute behavior, a two-dimensional numerical transport model was created to investigate solute transport under two hydrogeologic conditions:

1. homogeneous hydraulic conductivity in porous subsurface medium and
2. heterogeneous hydraulic conductivity in a fractured medium.

Table 2 describes the physical parameters used in the saturated homogenous porous medium for the numerical model of flow and transport. Except for increasing the hydraulic conductivity within the fracture, all other parameters are the same as in the homogenous model.

**Table 2:** **Physical parameters used in the homogenous model for flow and transport**

Parameter	Value	Description	Units
R	2.537e-8	Vertical recharge	m/s
N	0.15	Effective porosity	-
alpha_L	0.5	Longitudinal dispersivity	m^2^/s
alpha_T	0.005	Transverse dispersivity	m^2^/s
K1	3.17e-5	Hydraulic conductivity	m/s
C_in	2.22e8 (Co-60)	Initial activity concentration	Bq/m^3^
	5.66e12 (Cs-137)		
	3.5e7 (Am-241)		
D_m	2.78e6 (Co-60)	Effective diffusion coefficient	m^2^/s
	2.54e-9 (Cs-137)		
	3.17e-9 (Am-241)		
	5.27 (Co-60)	Radioactive half-life	Years
	30 (Cs-137)		
	432 (Am-241)		

### Model simulation results

Throughout the models, the amount of contaminant is shown by the colour bar. The activity concentration degree is indicated by the various colours, with red indicating an intense concentration.

### Evolution of ^60^Co

**Figure 5:**
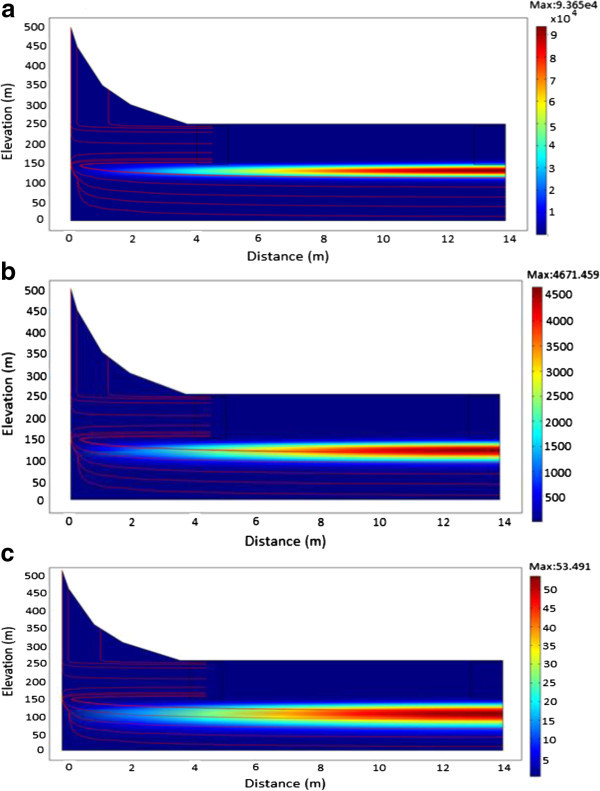
**A plot showing the migration of** ^**60**^ **Co. a**: From time of release to 250 years. **b**: From time of release to 500 years. **c**: From time of release to 1000 years

**Figure 6:**
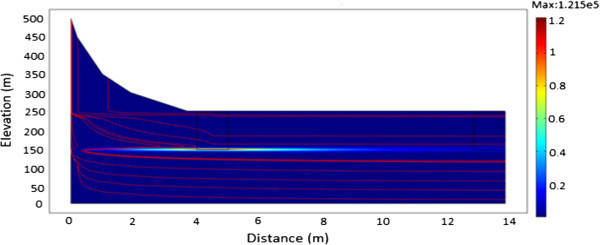
**A plot of** ^**60**^ **Co showing the effect of increased conductivity after 100 years.**

Radionuclide movement (streamline plot) and concentration estimates (surface plots) of ^60^Co simulated at various times are illustrated in Figures 5a to 6. ^60^Co has a half-life of 5.27 years and an initial inventory of 2.22 × 10^8^ Bq/m^3^. From the simulations carried out, it was evident that, for a homogenous porous medium in a low conductivity zone, ^60^Co contaminant source diffuses with the groundwater which has a flow velocity of 3.834 × 10^-8^ m/s. It was observed that, the radionuclide migrates in the same direction as the groundwater flow velocity while at the same time undergoing radioactive decay. However, when the hydraulic conductivity was increased (Figure 6) to account for heterogeneity within the system; it was observed that ^60^Co radionuclide plume does not rise towards the surface. Instead, the plume is diluted into deeper groundwater flow systems as it decayed away. This may be attributed to the short radioactive half-life as well as the high conductivity zone exhibited by the fractures.

**Figure 7:**
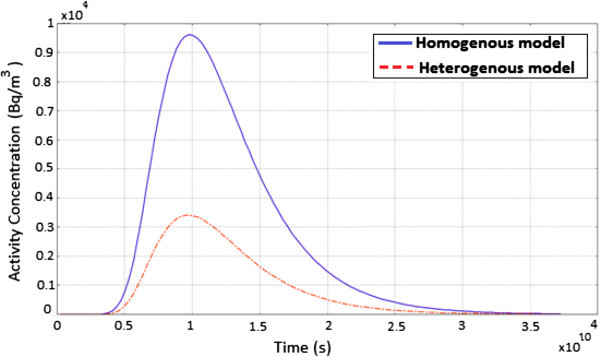
**A graph showing the evolution of** ^**60**^ **Co activity concentration as a function of time.**

Figure 7 shows the evolution of ^60^Co contaminant concentration as a function of time. Here, the diffusion process takes place much longer which is accounted for by the widening of the peak hence the radionuclide plume concentration appears broad in both models. For a homogenous porous medium, the concentration increase steadily until it reaches a maximum and then decreases exponentially. The high value of the initial activity concentration together with the diffusion process taking place, explains why the observed radionuclide plume extends over a very long range of time.

### Evolution of ^137^Cs

**Figure 8:**
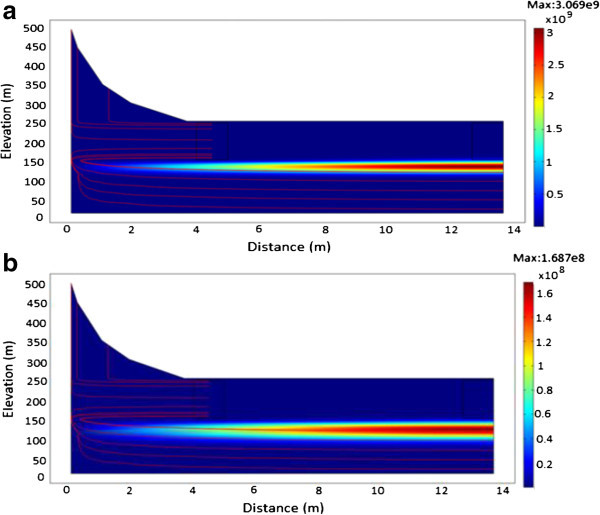
**A plot showing the migration of** ^**137**^ **Cs. a**: From time of release to 250 years. **b**: From time of release to 500 years.

^137^Cesium has a half-life of 30 years and an initial activity concentration of 5.66 × 10^12^ Bq/m^3^. Evolution of ^137^Cs contaminant source are shown in Figure 8a and b at various times (t = 250 years to t = 1000 years). In this case, ^137^Cs is migrating in a low conductivity medium causing the radionuclide to be deposited onto sediments which sinks steeply downwards through the groundwater flow system. The contaminant source diffuses with the flowing groundwater which has a measured flow velocity of 3.834 × 10^-8^ m/s. The flow velocity readily moves with ^137^Cs radionuclide in water causing contamination of groundwater.

**Figure 9:**
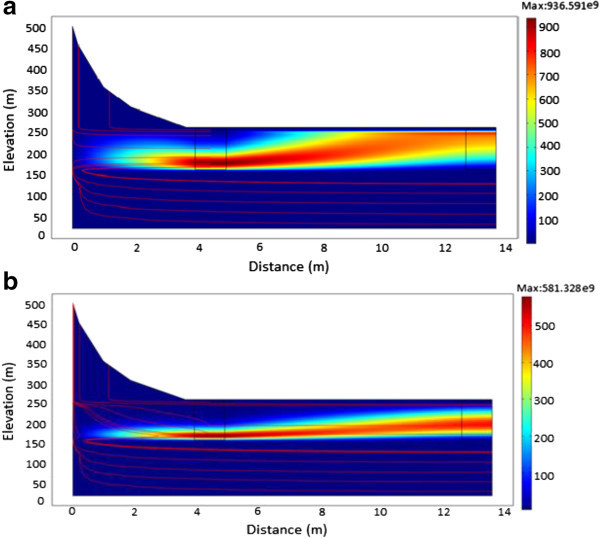
**A plot of** ^**137**^ **Cs showing the effect of increased conductivity. a**: After 40 years. **b**: After 60 years.

Figure 9a and b show the influence on radionuclide migration when the hydraulic conductivity is increased. After 40 years (Figure 9a) a fraction of its initial inventory would have undergone radioactive decay. Therefore in the presence of fractures, the increase in the hydraulic conductivity would provide preferential pathways for the flow velocity leading to an increase in mobility for the migrating radionuclide. The flow in this case causes the radionuclide plume to rise towards the surface which may finally discharge into wells, rivers and lakes endangering the environment, population and biota. Figure 9b shows that, radioactive decay, diffusive flux and the high hydraulic conductivity continues to have an effect in the direction and migration of the radionuclide source. These parameters tend to reduce the plume concentration as well as cause the radionuclide source to move away from the surface towards deeper groundwater flow systems contaminating groundwater resources.

**Figure 10:**
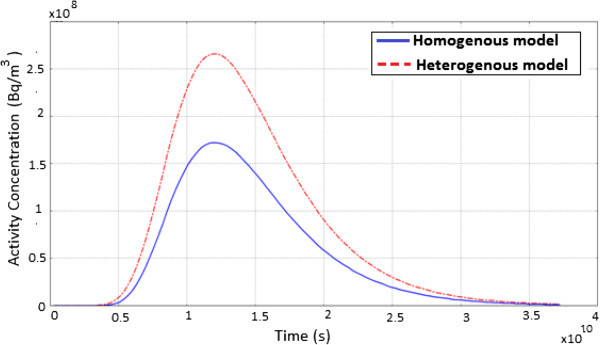
**A graph showing the evolution of** ^**137**^ **Cs activity concentration as a function of time.**

Figure 10 shows the evolution of ^137^Cs contaminant concentration as a function of time for the homogenous and heterogeneous model. For ^137^Cs, the radionuclide plume concentration appears broad suggesting that, the diffusion process takes place much longer which is accounted for by the widening of the peak. Also, the concentration increase steadily until it reaches a maximum and then decreases exponentially. The variation in the curves is a result of the presence of fractures which creates preferential pathways accounting for the high mobility of the radionuclide in the heterogeneous model.

### Evolution of ^241^Am

**Figure 11:**
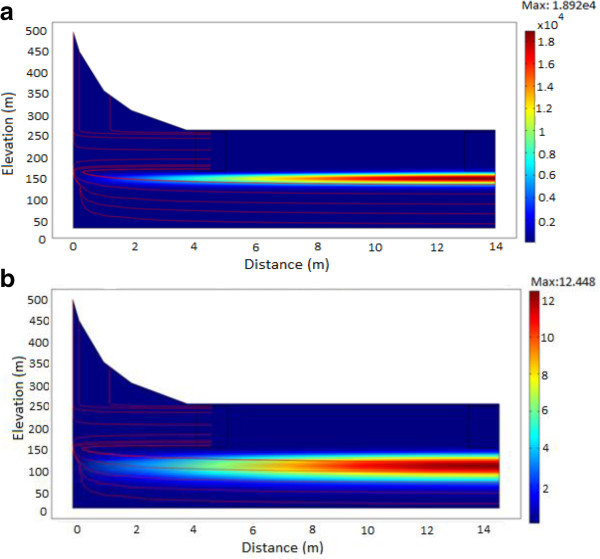
**A plot showing the migration of** ^**241**^ **Am. a**: From time of release to 250 years. **b**: From time of release to 1000 years.

The simulation was implemented for ^241^Am contaminant source with an initial activity concentration of 3.5 × 10^7^ Bq/m^3^, a half-life of 432 years and a flow velocity of 3.534 × 10^-8^ m/s. Figure 11a and b shows evolution of ^241^Am radionuclide activity concentration at various times in a homogenous porous medium having a low hydraulic conductivity of 3.17 × 10^-5^ m/s. From the simulations, ^241^Am was observed to sink steeply downward into the groundwater flow system after 200 years. At 250 years (Figure 11a), it continued to steeply decline into the groundwater flow system until about 1000 years (Figure 11b). Given that the value of the flow rate is low due to the low value of the hydraulic conductivity, transport of the radionuclide was mainly dominated by diffusion.

**Figure 12:**
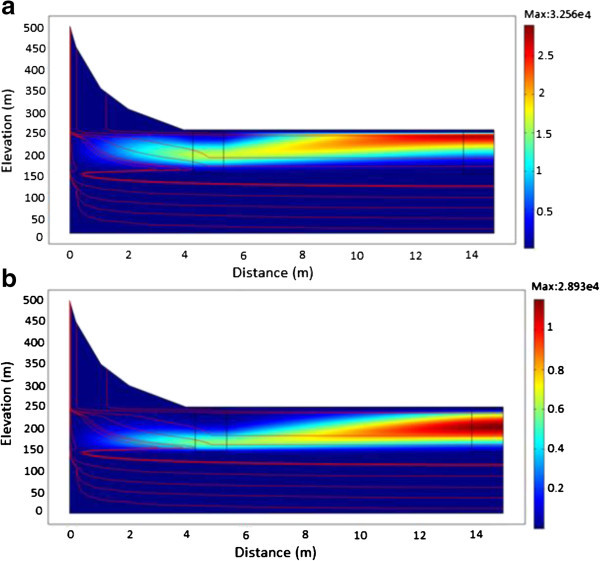
**A plot of** ^**241**^ **Am showing the effect of increased conductivity. a**: After 40 years. **b**: After 60 years.

Figure 12a to b shows the results obtained with an increase in hydraulic conductivity and its influence on the migration of ^241^Am radionuclide. From the simulations, groundwater moves with a flow velocity of 13.606 m/s. After 60 years (Figure 12a and b), the radionuclide source will not have undergone any appreciable decay process because of the long half-life (t_1/2_ = 432 years). Hence, majority of the radionuclide would appear to move towards the surface due to the high conductivity layer. In addition, lateral gradients can cause rapid rise of water-table leading to the possibility that the movement of ^241^Am attributable to diffusion is faster than the rate at which groundwater is moving. The reduction in the initial activity concentration of ^241^Am from 3.5 × 10^7^ Bq/m^3^ to 3.256 × 10^4^ Bq/m^3^ can be attributed to mass loss by the diffusive flux.

**Figure 13:**
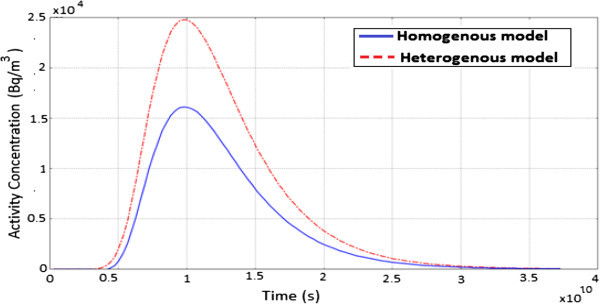
**A graph showing the evolution of** ^**241**^ **Am activity concentration as a function of time.**

Figure 13 shows the evolution of ^241^Am activity concentration as a function of time for a homogenous and heterogeneous model. The nature of the peak in both models suggests that, advection influences the distribution moderately with diffusion being the dominant transport mechanism. It is noticeable that, the peak concentration does not reach the initial activity value because of mass loss by radioactive decay. Similarly, the concentration reaches a maximum and then decreases exponentially. ^241^Am activity concentration extends over a very long range of time due to the high value of the initial activity, the long half-life and the diffusion process taking place.

## Conclusions

The migration of three radionuclides namely, ^60^Co, ^137^Cs and ^241^Am have been simulated using a two-dimensional finite element numerical model code (Comsol Multiphysics).

Neglecting heterogeneity, simulated results showed that, all three radionuclides (^60^Co, ^137^Cs, ^241^Am) within the low conductivity medium sunk steeply downward into the groundwater flow system by diffusing into the flowing groundwater. This caused the flow velocity to move readily with the radionuclide source causing contamination of groundwater resources.

In the presence of fractures, preferential pathways were created which gave rise to a rapid increase of the water-table and this caused the flow velocity to sweep the radionuclides with medium (^137^Cs) to long (^241^Am) half-life toward the surface endangering human population, the environment and biota.

For ^60^Co, the plume was not noticeably seen at the surface even in the presence of high hydraulic conductivity but was rather diluted into deeper groundwater flow systems as it decayed away. This was attributed to the short radioactive half-life.

The results obtained showed contamination to be more sensitive to variations in hydraulic conductivity as a result of the heterogeneity considered within the domain. However, impact on groundwater was still inevitable.

## Recommendation

It is recommended that, proper structural geological mapping including the use of stereograms should be made to be able to determine the fractures before radioactive waste is disposed of in an area.

## References

[CR1] Darko PK, Barnes EA, Sekpey NK (1995). Groundwater Assessment of the Accra Plains, Water Resources Research Institute (CSIR), Accra, Ghana.

[CR2] Essel P, Adjei-Kyereme Y, Akortia E, Abdallah M, Dawood A, Asomadu-Sakyi G, Nyarku M, Glover EET (2011). Ghana Atomic Energy Commission, Technical Report, Post-Closure Safety Assessment for Borehole Disposal of Disused Sealed Sources in Ghana.

[CR3] Junner NR, Bates DA (1995). Reports on the geology and hydrology of the coastal area east of the Akwapim Range, Gold Coast Geological Survey Memoir, No.7.

[CR4] Muff R, Efa E (2006). Environmental and Engineering Geology for Urban Planning in Accra and Tema, Ghana Geological Survey Department Bulletin No. 55.

